# Lessons from the COVID-19 pandemic: the unequal burden of COVID-19 on vulnerable populations in the Brazilian Central-West

**DOI:** 10.1590/0102-311XEN199623

**Published:** 2024-08-19

**Authors:** Kamila Cardoso dos Santos, Grazielle Rosa da Costa e Silva, Winny Éveny Alves Moura, Larissa Silva Magalhães, Bruno Vinícius Diniz e Silva, Gabriel Francisco da Silva, Lívia Melo Villar, Karlla Antonieta Amorim Caetano, Megmar Aparecida dos Santos Carneiro, Catalina Lopez-Quintero, Robert L. Cook, Krishna Vaddiparti, Sheila Araujo Teles, Regina Maria Bringel Martins

**Affiliations:** 1 Faculdade de Enfermagem, Universidade Federal de Goiás, Goiânia, Brasil.; 2 Instituto de Patologia Tropical e Saúde Pública, Universidade Federal de Goiás, Goiânia, Brasil.; 3 Instituto Oswaldo Cruz, Fundação Oswaldo Cruz, Rio de Janeiro, Brasil.; 4 Department of Epidemiology, University of Florida, Gainesville, U.S.A.

**Keywords:** COVID-19, Vulnerable Populations, Socioeconomic Factors, COVID-19, Populações Vulneráveis, Fatores Socieconômicos, COVID-19, Poblaciones Vulnerables, Factores Socioeconómicos

## Abstract

This study aimed to estimate the prevalence and identify social factors and preventive strategies associated with the coronavirus disease 2019 (COVID-19) in socio and economically vulnerable people (recyclable waste pikers, immigrants/refugees, and homeless people) in Goiânia, Goiás State, Central-Western Brazil. A cross-sectional study was conducted from July 2020 to October 2020. COVID-19 positivity was defined as a positive total anti-SARS-COV-2 antibody test and/or RNA test for SARS-COV-2. Univariable and multiple regression analyses were performed to identify the variables associated with COVID-19. Of the 594 participants, 47.3% were recyclable waste pickers, 29.6% were immigrants/refugees, and 23.1% were homeless people. The positivity for SARS-CoV-2 RNA was 14.1%, whereas for anti-SARS-CoV-2 a total of 30.8% were positive, and 39.4% were positive for at least one COVID-19 marker. Among the 541 individuals, being immigrants/refugees, not wearing a surgical mask, and having three or more people sleeping in the same room were associated with SARS-CoV-2 infection, while using TV news as the main source of information about the pandemic was a protective predictor of COVID-19. This study revealed ethnic and socioeconomic inequalities in the prevalence of COVID-19 among impoverished people in Brazil. Additionally, a high prevalence of COVID-19 was detected in all three groups. Developing new strategies to combat and prevent communicable diseases affecting this population is essential for mitigating future and ongoing pandemics.

## Resumo

Este estudo teve como objetivo estimar a prevalência da COVID-19 e identificar fatores sociais e estratégias preventivas associadas a essa doença em pessoas em situação de vulnerabilidade socioeconômica (catadores de lixo, imigrantes/refugiados e moradores de rua) em Goiânia, Estado de Goiás, na região central do Brasil. Trata-se de um estudo transversal realizado de julho a outubro de 2020. Foram considerados positivos para a covid-19 indivíduos que apresentaram um teste de anticorpos anti-SARS-COV-2 e/ou teste de RNA para SARS-COV-2 com resultado positivo. Foram realizadas análises de regressão univariável e múltipla para identificar as variáveis associadas à COVID-19. Dos 594 participantes, 47,3% eram catadores de lixo reciclável, 29,6% eram imigrantes/refugiados e 23,1% eram moradores de rua. A positividade foi de 14,1% para o RNA do SARS-CoV-2, 30,8% para o total de anti-SARS-CoV-2 e 39,4% para pelo menos um marcador da COVID-19. Entre os 541 indivíduos, ser imigrante/refugiado, não usar máscara cirúrgica e ter três ou mais pessoas dormindo no mesmo quarto foram fatores associados à infecção por SARS-CoV-2, enquanto confiar em informações sobre a pandemia, principalmente aquelas transmitidas por noticiários de TV, foi um indicador de proteção contra a COVID-19. Este estudo revelou desigualdades étnicas e socioeconômicas na prevalência da COVID-19 entre pessoas de baixa renda no Brasil. Além disso, uma alta prevalência de COVID-19 foi detectada nos três grupos. O desenvolvimento de novas estratégias para combater e prevenir doenças transmissíveis que afetam essa população é essencial para mitigar pandemias futuras e atuais.

## Resumen

Este estudio tuvo como objetivo estimar la prevalencia del COVID-19 e identificar los factores sociales y las estrategias preventivas asociadas a esta enfermedad en personas en situación de vulnerabilidad socioeconómica (recolectores de basura, inmigrantes/refugiados y personas sin hogar) en Goiânia, Estado de Goiás, en la región central de Brasil. Se trata de un estudio transversal realizado de julio a octubre de 2020. Las personas que tuvieron una prueba de anticuerpos anti-SARS-CoV-2 positiva y/o una prueba de ARN para SARS-CoV-2 se consideraron positivas para el COVID-19. Se realizaron análisis de regresión univariante y múltiple para identificar las variables asociadas al COVID-19. De los 594 participantes, el 47,3% eran recicladores; el 29,6% inmigrantes/refugiados; y el 23,1% eran personas sin hogar. La positividad fue del 14,1% para el ARN del SARS-CoV-2; del 30,8% para el total de anti-SARS-CoV-2; y del 39,4% para al menos un marcador del COVID-19. De los 541 participantes, ser inmigrante/refugiado, no usar mascarilla quirúrgica y tener tres o más personas que comparten la misma habitación fueron los factores asociados con la infección por SARS-CoV-2, mientras que confiar en la información sobre la pandemia, especialmente transmitidas por los noticiarios, fue un indicador de protección contra esta enfermedad. Este estudio reveló las desigualdades étnicas y socioeconómicas en la prevalencia del COVID-19 entre las personas de bajos ingresos en Brasil. Además, se detectó una alta prevalencia del COVID-19 en los tres grupos. El desarrollo de nuevas estrategias para combatir y prevenir las enfermedades transmisibles que afectan a esta población es fundamental para mitigar las pandemias futuras y actuales.

## Introduction

The coronavirus disease 2019 (COVID-19) pandemic represents the most significant public health crisis of the 21st century ^1^. It is caused by severe acute respiratory syndrome coronavirus 2 (SARS-CoV-2), which is transmitted primarily by droplets and aerosols generated during coughing or sneezing od infected individuals. Additionally, indirect transmission can occur via contact with objects or surfaces contaminated by respiratory secretions of infected individuals ^2^ ^,^ ^3^. Generally, SARS-CoV-2 infection starts with mild respiratory symptoms, but it can quickly progress to a severe respiratory syndrome. Available evidence indicates that severe symptoms with worse prognoses are more frequent in men, older adults, individuals with comorbidities, and immunocompromised individuals ^4^. In Brazil, SARS-CoV-2 infection initially affected high-income people returning from overseas. However, it quickly spread to other groups, overwhelmingly affecting poorer populations ^5^.

According to the *Human Development Report 2021/2022* ^6^, at least 1.3 billion people worldwide live in multidimensional poverty, facing health, education, and food deprivation, among others. The pandemic amplified this scenario, increasing the number of people living in extreme poverty (surviving on less than USD 1.90/day) for the first time in over two decades ^6^.

Economic and political crises in Brazil have resulted in high unemployment rates, consequently increasing the figures of informal workers, such as recyclable waste pickers and homeless people. Moreover, there has been a large influx of immigrants in recent years, with more than 50,000 people, mainly from Venezuela, requesting refugee status in 2022 ^7^. Most of these emergent populations live in extreme poverty, in hazardous conditions and crowded housing facilities, socially isolated, and without basic sanitation. Additionally, they are often exposed to high-stress jobs with low wages and have difficulty accessing public healthcare services, making them vulnerable to infectious diseases such as COVID-19 ^8^ ^,^ ^9^. Therefore, COVID-19 has been recognized as a syndemic, in which the complex interaction between SARS-CoV-2 infection and existing social, economic, and health disparities can increase the chances of higher positivity rates and worse outcomes ^10^.

The increasing global environmental changes, including deforestation and urbanization, contribute indirectly to the emergence of new viral agents ^11^. Therefore, it is important to elucidate the extent to which SARS-CoV-2 affected the marginalized groups of developing countries, such as Brazil, and the measures undertaken to face the pandemic. Understanding the epidemiology of this pandemic in low-income and vulnerable populations will support appropriate handling of similar outbreaks affecting the above-mentioned groups in the future. In this study, we aimed to estimate the prevalence of COVID-19 and identify social factors and preventive strategies adopted against COVID-19 in socially and economically vulnerable people (immigrants/refugees, recyclable waste pickers, and homeless individuals) in Goiânia, Goiás, State, Brazilian Central-West, during the first and second waves of COVID-19.

## Methodology

### Study design

A cross-sectional study was conducted from August 2020 to April 2021 among three socially and economically vulnerable populations: immigrants/refugees, recyclable waste pickers, and homeless people.

### Population and location

In Brazil, COVID-19 testing availability was limited. Therefore, the Federal University of Goiás developed a community project for no-cost SARS-CoV-2 screening. To support this effort, a strict biosafety infrastructure was created on the university premises. All safety protocols were followed, including reducing the crowding of participants. Thus, the research team successfully reached vulnerable populations in the community and conducted COVID-19 screening.

The population consisted of immigrants/refugees (80% Venezuelans), recyclable waste pickers, and homeless people from Goiânia, assisted by nongovernmental organizations (NGOs). During the data collection period, there were no data on COVID-19 prevalence in Brazil, and no vaccines were available. Therefore, we determined the cohort size as a minimum of 329 individuals, considering a hypothetical 5% prevalence, a 95% significance level (α < 0.05), a 3% precision, and a 2% effect design.

We included socially vulnerable individuals from Goiânia who were referred by NGOs, leaders, and government entities partnering in this project. We excluded individuals who were visibly under the influence of alcohol and/or illicit drugs and exhibited behaviors that jeopardized the team’s safety, or faced difficulties in answering to questions.

### Recruitment

In July 2020, the research team contacted NGO partners who were providing social assistance to immigrants/refugees, recyclable waste pickers, and homeless individuals in Goiânia, and disclosed the study purpose. All agreed to collaborate and were eager to participate in COVID-19 screening efforts. The representatives of the NGO then committed to invite eligible individuals to participate in the study, scheduling their data and sample collection, and providing their transportation to the location where data and samples were collected.

### Ethical aspects

This study was approved by the Ethics Committee of the Federal University of Goiás (n. 4,249,851) according to *Resolution n. 466/12*.

### Data and samples collection

All participants were interviewed using a questionnaire regarding demographic and social characteristics (sex, age, marital status, years of education, employment, and monthly household income), flu-like signs and symptoms in themselves or family since the beginning of the pandemic, and pandemic coping measures (related to their knowledge about the pandemic, hand hygiene, and use of mask). For immigrants/refugees, interviews were conducted with individuals who were fluent in Spanish and trained to adhere to all safety and research protocols. Interviews were conducted after explaining the project and obtaining written informed consent.

Blood samples (10mL) were collected and tested for total anti-SARS-CoV-2 antibodies using an electrochemiluminescence assay (Elecsys Anti-SARS-CoV-2 cobas. Roche; https://diagnostics.roche.com). 

Oropharyngeal and nasopharyngeal samples were collected using combined swabs (nasal/oral) to detect SARS-CoV-2 RNA. Viral RNA was extracted using the QIAamp Viral RNA Mini Kit (Qiagen; https://www.qiagen.com), and real-time quantitative polymerase chain reaction was performed using AgPath-IDTM One-Step RT-PCR Reagents (Thermo Fisher Scientific; https://www.thermofisher.com). Assays were performed using probes from the 2019-nCoV kit (Integrated DNA Technologies; https://www.idtdna.com/pages).

**Figure 1:**
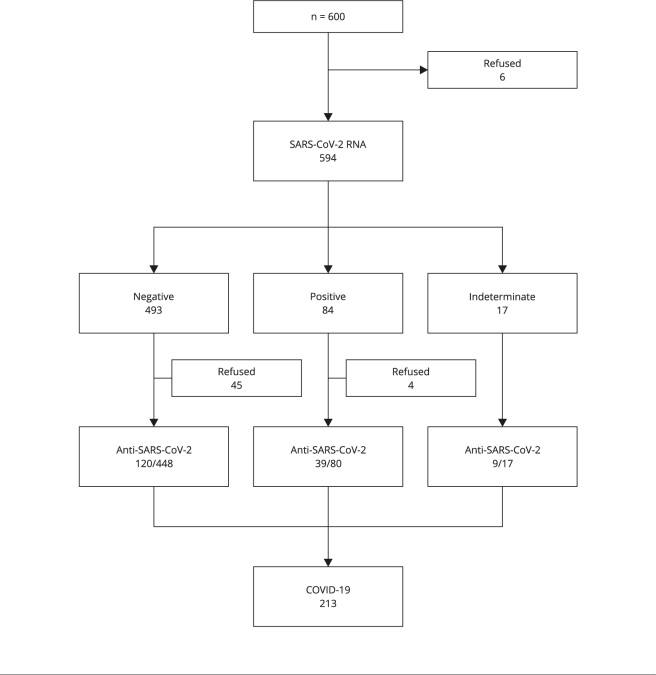
Detection of SARS-CoV-2 according to markers.

In this study, COVID-19 diagnosis was the dependent variable. Participants were classified as positive for COVID-19 if they tested positive for SARS-CoV-2 RNA and/or total anti-SARS-CoV-2 antibodies (Figure 1).

The independent variables included demographic and social characteristics, such as sex, age, marital status, years of education, employment, number of residents in the household, monthly household income, pandemic coping measures, COVID-19 symptoms in the family, knowledge of the pandemic, hand hygiene, and use of personal protective equipment.

### Data analysis

The interview data and laboratory results were analyzed using the SPSS version 20.0 software (https://www.ibm.com/). Prevalence was estimated using 95% confidence interval (95%CI). For each group, prevalence ratio (PR) and 95%CI were used to measure the strength of the association between COVID-19 diagnosis (outcome) and the independent variables. Variables associated with the outcome, with p < 0.20, were included in Poisson regression with a robust variance model ^12^. The significance level was set at 0.05. Pearson’s chi-squared goodness-of-fit test was used to assess the model adequacy.

## Results 

Six of the 600 participants refused oro-nasopharyngeal sample collection. Table 1 presents the socio-demographic characteristics of the 594 individuals included in this study. Of the total participants, 281 (47.3%) were recyclable waste pickers, 176 (29.6%) were immigrants/refugees, and 137 (23.1%) were homeless individuals. The median age was 35 years (interquartile range - IQR: 26-47), with a monthly income of BRL 1,100 (IQR: 1,000-2,000) and with 9 years of education (IQR: 6-12). Regarding sex, the majority were male individuals (314; 52.9%). Ninety-six individuals wer white, and 498 (83.8%) were non-white (313 were mixed-race, 147 were black, 13 were Asians, and 25 were indigenous). Most participants were single (65.6%) and had at least one COVID-19 symptom (flu-like symptoms) (60%).

**Table 1:** Sociodemographic characteristics of the 594 vulnerable people tested for SARS-CoV-2 markers during the first and second waves of the COVID-19 in Goiânia, Goiás State, Central-Western Brazil.

Category	n	%
Vulnerable population		
Recyclable waste pickers	281	47.3
Immigrants/Refugees	176	29.6
Homeless	137	23.1
Gender		
Male	314	52.9
Female	280	47.1
Race/Ethnicity		
White	96	16.2
Non-white	498	83.8
Marital status (no information: 9)		
Single/Divorced/Widowed	384	65.6
Married	201	31.4
COVID symptoms (flu-like symptoms) (no information: 6)		
No	235	40.0
Yes	353	60.0
Continuous variables	Median	Q1-Q3
Age (years)	35	26-47
Monthly income (BRL *)	1,100	1,000-2,000
Years of education	9	6-12

Q1-Q3: quartile 1 to quartile 3.

* BRL 5.64 was equivalent to USD 1.

**Figure 2:**
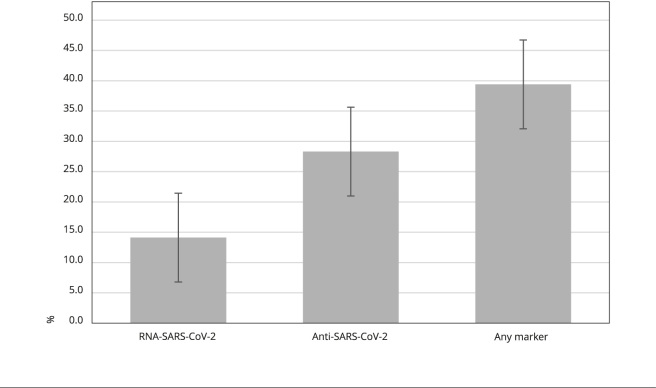
Prevalence of COVID-19 markers among vulnerable people.

Among the 594 individuals tested for SARS-CoV-2 RNA, 493 (83%) tested negative, 84 (14.14%) tested positive, and 17 (2.86%) received indeterminate results. Also, 545 participants were tested for total anti-SARS-CoV-2. Of them, 168 (30.8%) tested positive. Of 541 individuals, 39.4% showed positive results for at least one COVID-19 marker (Figures 1 and 2).

Table 2 shows the univariable analysis of sociodemographics and conditions of coping with the COVID-19 pandemic. In the 541 individuals, being an recyclable waste pickers (PR = 1.84; 95%CI: 1.17-2.88), being an immigrant/refugee (PR = 4.01; 95%CI: 2.63-6.13), not using surgical masks (PR = 1.50; 95%CI: 1.04-2.18), and sharing the same room with more than three people (PR = 1.74; 95%CI: 1.40-2.17) were associated with COVID-19-positive results. These variables, along with age (PR = 0.99; 95%CI: 0.95-1.00), marital status (PR = 1.16; 95%CI: 0.93-1.48), working status during the pandemic (PR = 1.18; 95%CI: 0.94-1.48), COVID symptoms in the family (flu-like symptoms) (PR = 1.16; 95%CI: 0.94-1.44), and relying on TV news as the main source of information about the pandemic (PR = 0.84; 95%CI: 0.67-1.05) were included in the multiple Poisson regression.

**Table 2:** Univariable analysis of sociodemographics and coping strategies according to COVID-19 markers in 541 vulnerable participants in Goiânia, Goiás State, Central-Western Brazil.

Parameter	COVID-19+	COVID-19-	p-value *	PR (95CI%)
	n (%)	n (%)		
Age (years) [median (Q1-Q3)]	32 (23-45)	35 (27-46)	0.118	0.99 (0.95-1.00)
Monthly income (BRL *) [median (Q1-Q3)]	1,100 (1,000-2,000)	1,100 (1,000-2,000)	0.802	1.00 (1.00-1.00)
Years of education [median (Q1-Q3)]	9 (6-12)	9 (6-12)	0.347	1.01 (0.98-1.04)
Vulnerable population				
Homeless	19 (16.8)	94 (83.2)		1.00
Recyclable waste picker	80 (30.9)	179 (69.1)	0.008	1.84 (1.17-2.88)
Immigrants/Refugees	114 (67.5)	55 (32.5)	< 0.001	4.01 (2.63-6.13)
Gender				
Male	108 (39.6)	165 (60.4)	0.928	0.99 (0.80-1.22)
Female	105 (39.2)	163 (60.8)		
Race/Ethnicity				
White	31 (35.6)	56 (64.4)	0.448	1.13 (0.83-1.53)
Non-white	182 (40.1)	272 (59.9)		
Marital status (no information: 9)				
Single/Divorced/Widowed	129 (37.2)	218 (62.8)	0.184	1.16 (0.93-1.43)
Married	80 (43.0)	106 (57.0)		
Worked during the pandemic				
No	60 (46.2)	70 (53.8)	0.163	1.18 (0.94-1.48)
Yes	133 (39.2)	206 (60.8)		
Cloth mask				
Yes	206 (40.2)	306 (59.8)	0.772	1.09 (0.62-1.92)
No	7 (43.8)	9 (56.2)		
Surgical mask				
Yes	22 (28.2)	56 (71.8)	0.031	1.50 (1.04-2.18)
No	191 (42.4)	259 (57.6)		
N-95 mask				
Yes	5 (27.8)	13 (72.2)	0.317	1.47 (0.69-3.12)
No	302 (59.2)	208 (40.8)		
Hand wash				
Yes	149 (39.7)	223 (60.3)	0.797	0.97 (0.77-1.22)
No	64 (38.6)	102 (61.4)		
Number of people sleeping in the same room				
Only one	115 (33.7)	226 (66.3)		1.00
Two people	37 (40.7)	54 (59.3)	0.206	1.21 (0.90-1.61)
Three or more people	61 (58.7)	43 (41.3)	< 0.001	1.74 (1.40-2.17)
COVID symptoms in family (flu-like symptoms)				
No	107 (35.7)	193 (64.3)	0.171	1.16 (0.94-1.44)
Yes	83 (43.7)	107 (56.3)		
TV news as the main source of information about the pandemic				
No	58 (45.0)	71 (55.0)	0.131	0.84 (0.67-1.05)
Yes	155 (37.7)	256 (62.3)		
Radio broadcasts as the main source of information about the pandemic				
No	175 (38.8)	276 (61.2)	0.483	1.10 (0.84-1.44)
Yes	38 (42.7)	51 (57.3)		
Social media as the main source of information about the pandemic				
No	141 (39.9)	72 (38.5)	0.746	0.96 (0.77-1.20)
Yes	212 (60.1)	115 (61.5)		
Friends as the main source of information about the pandemic				
No	161 (40.0)	241 (60.0)	0.627	0.94 (0.74-1.20)
Yes	52 (37.7)	86 (62.3)		
Family as the main source of information about the pandemic				
No	171 (39.0)	267 (61.0)	0.688	1.05 (0.81-1.37)
Yes	42 (41.2)	60 (58.8)

95%CI: 95% confidence interval; PR: prevalence ratio; Q1-Q3: quartile 1 to quartile 3.

* Wald test;

** BRL 5.64 was equivalent to USD 1.

Being an immigrant/refugee (adjusted PR = 2.95; 95%CI: 1.76-4.94), not wearing a surgical mask (adjusted RP = 1.44; 95%CI: 1.00-2.07), and sharing the same room with three people or more (adjusted RP = 1.32; 95%CI: 1.04-1.69) remained independently associated with the outcome as risk factors. On the contrary, relying on TV news as the main source of information about the pandemic (adjusted RP = 0.80; 95%CI: 0.65-1.00) remained independently associated with the outcome as a protective factor (Table 3).

**Table 3:** Multiple Poisson regression analysis of variables associated with COVID-19 among vulnerable populations in Goiânia, Goiás State, Central-Western Brazil.

Parameter	p-value *	Adjusted PR (95%CI) **
Vulnerable population		
Homeless		1.00
Waste recycle pickers	0.149	1.49 (0.87-2.55)
Immigrants/Refugees	< 0.001	2.95 (1.76-4.94)
Surgical mask		
Yes		1.00
No	0.051	1.44 (1.00-2.07)
Number of people sleeping in the same room		
Only one		1.00
Two people	0.940	0.99 (0.72-1.36)
Three or more people	0.023	1.32 (1.04-1.69)
TV news as the main source of information about the pandemic		
No		1.00
Yes	0.046	0.80 (0.65-1.00)

95%CI: 95% confidence interval; PR: prevalence ratio.

* Wald test;

** Adjusted by population, age, marital status, worked during the pandemic, surgical mask, number of people sleeping in the same room, COVID symptoms in family, TV news as the main source of information about the pandemic.

## Discussion

Herein, we describe the prevalence of SARS-CoV-2 infection and the associated variables among immigrants/refugees, recyclable waste pickers, and homeless individuals in Central-Western Brazil. Notably, this study was conducted before the COVID-19 vaccination program. Therefore, the epidemiological data on COVID-19 in these vulnerable target groups are important to establish better strategies for preventive actions in future outbreaks.

At the onset of the pandemic in Brazil, Hallal et al. ^13^ conducted two successive nationwide serological household surveys in May and June 2020. They tested participants for total antibodies against SARS-CoV-2 using a lateral-flow point-of-care test and found that the pooled prevalence increased from 1.9% to 3.1% among cities, with 200 or more individuals tested in both surveys, highlighting accelerated viral dissemination. Furthermore, both surveys showed that the prevalence in the poorest people was twice as high as that of the richest people. Similarly, this study included people from the lowest layer of the population; 14.1% were already infected as determined by SARS-CoV-2 RNA positivity, and 30.8% had antibodies against SARS-CoV-2. These findings reinforce the uncontrolled viral dissemination in Brazil, particularly among the poorest , as well as the urgent need to promptly include socially and economically vulnerable groups in the target population.

We studied three groups of impoverished people living with limited social protection: immigrants/refugees, recyclable waste pickers, and homeless people. A high frequency of SARS-CoV-2 exposure was found in all of them. However, the disproportional viral dissemination among immigrants/refugees is noteworthy, as 67.5% had been exposed to the new coronavirus. These findings align with the epidemiological bulletin of the state of Roraima (Brazil-Venezuela border), which reported that over 80% of deaths caused by COVID-19 occurred among Venezuelan immigrants ^14^. Investigations conducted with immigrants in other countries have reinforced their higher social vulnerability. For example, in the United Kingdom, hospital death rates are higher among immigrants, accounting for 35% of all patients with COVID-19 in intensive care units ^15^. Even in the United States and Europe, hospitalization and death rates are higher among immigrant populations ^16^ ^,^ ^17^ ^,^ ^18^.

Most impoverished people live in overcrowded housing conditions with shared bedrooms or even beds, making it difficult to designate a separate room for everyone. Thus, infected, and non-infected individuals live in the same environment, increasing the chances of viral transmission and acquisition ^19^ ^,^ ^20^ ^,^ ^21^. In this study, individuals who reported sleeping with three or more individuals in the same bedroom were more likely to have a positive test for COVID-19.

A low prevalence of COVID-19 was observed among individuals who relayed on TV news as the main source of information about the pandemic. TV is the mode of communication with most publicity and participation of government representatives, including public health managers. In addition, television information is provided in an accessible and clear language, without the need for double-checking. Hence, traditional media is considered a reliable source of information and contributes to improving prevention behaviors ^22^ ^,^ ^23^.

During the study period, COVID-19 vaccination and treatment were not available. Therefore, non-pharmacological interventions, such as physical distancing, face masks, and hand hygiene were the primary measures to control and prevent the new coronavirus outbreak ^21^ ^,^ ^24^. Most participants reported using masks; however, most of them used cloth masks. Those who reported the use of surgical masks showed a lower frequency contracting COVID-19. Therefore, during epidemics, public managers must distribute free surgical masks for impoverished people as a strategy to mitigate the ongoing communicable disease.

This study had some limitations. We used a convenience sampling method and considered the three groups as a whole. Therefore, our data may not represent all the studied groups. Furthermore, the cross-sectional design provided limited evidence to support causality, although reverse causality was unlikely in this study. Despite these limitations, our data show epidemiological coherence and plausibility.

## Conclusion

We showed a high prevalence of COVID-19 among the three studied groups, with the most affected being immigrants/refugees, followed by the Brazilian recyclable waste pickers, and homeless people. In addition, not wearing a surgical mask and having three or more people sleeping in the same room were associated with SARS-CoV-2 infection, while relying on TV news as the main source of information about the pandemic was a protective predictor of COVID-19. Finally, these findings highlight the need for stakeholders to consider the specificities of socially vulnerable individuals to control and prevent emergent infections.

## References

[1] World Health Organization WHO coronavirus (COVID-19) dashboard..

[2] Cheng ZJ, Shan J (2020). 2019 Novel coronavirus where we are and what we
know. Infection.

[3] Kampf G, Todt D, Pfaender S, Steinmann E (2020). Persistence of coronaviruses on inanimate surfaces and their
inactivation with biocidal agents. J Hosp Infect.

[4] Fang X, Li S, Yu H, Wang P, Zhang Y, Chen Z (2020). Epidemiological, comorbidity factors with severity and prognosis
of COVID-19 a systematic review and meta-analysis. Aging.

[5] Magalhães JJF, Mendes RPG, Silva CTA, Silva SJR, Guarines KM, Pena L (2020). Epidemiological and clinical characteristics of the first 557
successive patients with COVID-19 in Pernambuco state, Northeast
Brazil. Travel Med Infect Dis.

[6] United Nations Development Programme (2022). Human Development Report 2021-22. Uncertain times, unsettled lives:
shaping our future in a transforming world.

[7] Junger da Silva G, Cavalcanti L, Lemos Silva S, Tonhati T, Lima Costa LF (2023). Refúgio em números 2023..

[8] Quinn SC, Kumar S (2014). Health inequalities and infectious disease epidemics a challenge
for global health security. Biosecur Bioterror.

[9] Mamelund SE, Dimka J (2021). Social inequalities in infectious diseases. Scand J Public Health.

[10] Horton R (2020). Offline COVID-19 is not a pandemic. Lancet.

[11] Oliveira T, Tegally H (2023). Will climate change amplify epidemics and give rise to
pandemics. Science.

[12] Barros AJ, Hirakata VN (2003). Alternatives for logistic regression in cross-sectional studies
an empirical comparison of models that directly estimate the prevalence
ratio. BMC Med Res Methodol.

[13] Hallal PC, Hartwig FP, Horta BL, Silveira MF, Struchiner CJ, Vidaletti LP (2020). SARS-CoV-2 antibody prevalence in Brazil results from two
successive nationwide serological household surveys. Lancet Glob Health.

[14] Secretaria de Estado da Saúde de Roraima, Governo do Estado de
Roraima (2022). Cenário epidemiológico de Roraima.. Boletim Epidemiológico sobre a Doença pelo Coronavírus 2019
(COVID-19).

[15] Platt L, Warwick R (2020). Are some ethnic groups more vulnerable to COVID-19 than others?.

[16] Rossi PG, Marino M, Formisano D, Venturelli F, Vicentini M, Grilli R (2020). Characteristics and outcomes of a cohort of COVID-19 patients in
the Province of Reggio Emilia, Italy. PLoS One.

[17] Islamoska S, Petersen JH, Benfield T, Norredam M (2022). Socioeconomic and demographic risk factors in COVID-19
hospitalization among immigrants and ethnic minorities. Eur J Public Health.

[18] Ross J, Diaz CM, Starrels JL (2020). The disproportionate burden of COVID-19 for immigrants in the
Bronx, New York. JAMA Intern Med.

[19] Deng W, Sun Y, Yao X, Subramanian K, Ling C, Wang H (2022). Masks for COVID-19. Adv Sci.

[20] Tabatabaeizadeh S-A (2021). Airborne transmission of COVID-19 and the role of face mask to
prevent it a systematic review and meta-analysis. Eur J Med Res.

[21] Talic S, Shah S, Wild H, Gasevic D, Maharaj A, Ademi Z (2021). Effectiveness of public health measures in reducing the incidence
of covid-19, SARS-CoV-2 transmission, and covid-19 mortality systematic
review and meta-analysis. BMJ.

[22] Niu Z, Qin Z, Hu P, Wang T (2022). Health beliefs, trust in media sources, health literacy, and
preventive behaviors among high-risk Chinese for covid-19. Health Commun.

[23] Shafiq M, Elharake JA, Malik AA, McFadden SM, Aguolu OG, Omer SB (2021). COVID-19 sources of information, knowledge, and preventive
behaviors among the US adult population. J Public Health Manag Pract.

[24] Stefanati A, D'Anchera E, Motoli F, Savio M, Gabutti G (2021). Evaluation and review of preventive measures applied during
COVID-19 pandemic: strategies adopted by European countries.. J Prev Med Hyg.

